# How much is needed? Patient exposure and curricular education on medical students’ LGBT cultural competency

**DOI:** 10.1186/s12909-020-02381-1

**Published:** 2020-12-04

**Authors:** Dustin Z. Nowaskie, Anuj U. Patel

**Affiliations:** 1 Department of Psychiatry, Indiana University School of Medicine, 355 W. 16th St, #2364, Indianapolis, IN 46202 USA; 2 University of Michigan Medical School, Ann Arbor, MI USA

**Keywords:** Attitudes, Cultural competency, Knowledge, LGBT, Medical education, Patients, Preparedness, Student

## Abstract

**Background:**

For medical students, providing exposure to and education about the lesbian, gay, bisexual, and transgender (LGBT) patient population are effective methods to increase comfort, knowledge, and confidence in caring for LGBT people. However, specific recommendations on the number of patient exposures and educational hours that relate to high LGBT cultural competency are lacking.

**Methods:**

Medical students (*N* = 940) at three universities across the United States completed a survey consisting of demographics, experiential variables (i.e., number of LGBT patients and LGBT hours), and the 7-point Likert LGBT-Development of Clinical Skills Scale (LGBT-DOCSS). LGBT-DOCSS scores were stratified by 1-point increments, and experiential variable means were computed per each stratification to characterize the mean LGBT patients and hours of medical students with higher scores and those with lower scores.

**Results:**

Medical students reported caring for some LGBT patients annually (M = 6.02, SD = 20.33) and receiving a low number of annual LGBT curricular hours (M = 2.22, SD = 2.85) and moderate number of annual LGBT extracurricular hours (M = 6.93, SD = 24.97). They also reported very high attitudinal awareness (M = 6.54, SD = 0.86), moderate knowledge (M = 5.73, SD = 1.01), and low clinical preparedness (M = 3.82, SD = 1.25). Medical students who cared for 35 or more LGBT patients and received 35 or more LGBT total hours reported significantly higher preparedness and knowledge.

**Conclusions:**

Medical students have shortcomings in LGBT cultural competency and limited LGBT patient exposure and education. To improve LGBT cultural competency, medical schools and accrediting bodies should consider providing medical students with at least a total of 35 LGBT patient contacts and 35 LGBT education hours (10 h of required curricular education and 25 h of supplemental education).

**Supplementary Information:**

The online version contains supplementary material available at 10.1186/s12909-020-02381-1.

## Background

Significant health disparities exist within the lesbian, gay, bisexual, and transgender (LGBT) population. Namely, LGBT people endure higher rates of poor physical and mental health, activity limitations, and chronic disease compared to their cisgender, heterosexual peers [1–3]. In addition, discrimination in healthcare encounters for this patient population is reportedly as high as 20% [4] and has been shown to occur in forms such as medication refusal as well as verbal and physical violence [5]. These acts can ultimately lead LGBT patients to avoid essential healthcare, and in turn, cause an exacerbation of existing health disparities [5].

Training programs that provide exposure to LGBT patients can increase medical students’ comfort levels in caring for this population [6]. Likewise, LGBT education programs can increase knowledge [6] and confidence in clinical assessments [7] of LGBT people among medical students. Over the past decade, many medical schools have made efforts to include LGBT-specific healthcare topics in their curricula. However, there remains significant biases [8] and varying levels of preparedness [9] among these learners, which may be a result of variability in educational initiatives with regards to curricular content, curricular hours, and patient exposure. With respect to the transgender population, medical students have reported feeling significantly less knowledgeable and comfortable treating transgender patients compared to lesbian, gay, and bisexual (LGB) patients [10, 11]. Medical students have also expressed a desire to improve their communication skills and clinical practices with transgender care [11]. While Obedin-Maliver et al. have characterized the wide range of LGBT education delivered in medical schools in the United States and Canada [12], specific recommendations on the number of patient exposures and educational hours that can lead to high LGBT cultural competency are lacking. Such recommendations could allow for a standardized approach to LGBT curricular education for medical schools.

For these reasons, we undertook a multicenter study to characterize medical students’ LGBT cultural competency. We aimed to investigate the relationship between medical students’ cultural competency and experiential variables (i.e., the amount of LGBT patient exposure and curricular education they received). We hypothesized that medical students would report high LGBT attitudinal awareness and lower knowledge and clinical preparedness. We posited that medical students would feel less prepared to clinically assess transgender patients compared to LGB patients. Further, we hypothesized that medical students who reported higher LGBT cultural competency would also report more LGBT patient exposure and curricular education. With the data presented herein, we aimed to characterize the LGBT experientials of higher-competent medical students, and thereby propose these amounts as specific educational recommendations which could promote high LGBT cultural competency for medical students.

## Methods

### Instrument

As part of a larger study [13], an anonymous, cross-sectional, self-reporting online survey comprised of 28 items was utilized for data collection. This survey consisted of the LGBT-Development of Clinical Skills Scale (LGBT-DOCSS) [14] as well as demographic and experiential variables. The demographic variables collected were age, gender identity, sexual orientation, race, ethnicity, university, and level of training (Table 1). Three items addressed experiential variables: the number of “LGBT patients” the medical students had worked with or cared for and the number of “LGBT curricular hours” and “LGBT total hours” they had received at their current school and ever, respectively. Medical students were also asked if there was anything else that they would like to share regarding LGBT healthcare.

**Table 1:** Demographic and experiential variables (*N* = 940)^a^

	M (SD) or n (%)
Age	25.49 (2.90)
LGBT experientials	
Patients	13.74 (27.68)
Annual patients	6.02 (20.33)
Curricular hours	5.32 (7.74)
Annual curricular hours	2.22 (2.85)
Extracurricular hours	12.46 (43.01)
Annual extracurricular hours	6.93 (24.97)
Gender identity	
Cisgender man	344 (36.6%)
Cisgender woman	586 (62.3%)
Non-binary	4 (0.4%)
Transgender man	2 (0.2%)
Transgender woman	2 (0.2%)
Other^b^	2 (0.2%)
Sexual orientation	
Bisexual	80 (8.5%)
Gay	48 (5.1%)
Heterosexual	769 (81.8%)
Lesbian	14 (1.5%)
Queer	15 (1.6%)
Other^b^	14 (1.5%)
Race	
Asian/Asian American	158 (16.8%)
Black/African American	30 (3.2%)
White/Caucasian	674 (71.7%)
Other^b^	78 (8.3%)
Ethnicity	
Hispanic or Latino	67 (7.1%)
Not Hispanic or Latino	873 (92.9%)
University	
University #1	392 (41.7%)
University #2	257 (27.3%)
University #3	291 (31.0%)
Level of training	
First year	244 (26.0%)
Second year	250 (26.6%)
Third year	226 (24.0%)
Fourth year	195 (20.7%)
Fifth year and above	22 (2.3%)

Abbreviations: *LGBT* lesbian, gay, bisexual, and transgender

^a^N = 940 for all variables except: age (*n* = 939), LGBT patients (*n* = 871), LGBT curricular hours (*n* = 929), LGBT extracurricular hours (*n* = 926), and level of training (*n* = 937)

^b^For “other” categories:

• gender identity: other (*n* = 2)

• sexual orientation: asexual (*n* = 3), asexual & demisexual (*n* = 1), asexual & queer (*n* = 1), bisexual & heterosexual (*n* = 1), bisexual & queer (*n* = 2), gay & queer (*n* = 2), heterosexual & queer (*n* = 2), heterosexual & questioning (*n* = 1), and questioning (*n* = 1)

• race: Alaska Native & American Indian (*n* = 1), Albanian & White/Caucasian (*n* = 1), American Indian (*n* = 2), American Indian & Black/African American & White/Caucasian (*n* = 1), American Indian & White/Caucasian (*n* = 8), Ashkenazi Jewish (*n* = 1), Asian/Asian American & Middle Eastern & White/Caucasian (*n* = 1), Asian/Asian American & White/Caucasian (*n* = 25), Asian/Asian American & White/Caucasian & other (*n* = 1), Black/African American & White/Caucasian (*n* = 9), Filipino/Haitian (*n* = 1), French Creole & White/Caucasian (*n* = 1), Jewish (*n* = 1), MENA (*n* = 1), Mexican (*n* = 1), Middle Eastern (*n* = 2), Middle Eastern & North African (*n* = 1), mixed race (*n* = 1), Native Hawaiian (*n* = 2), other (*n* = 15), Pacific Islander & White/Caucasian (*n* = 1), and White/Caucasian & other (n = 1)

The LGBT-DOCSS is 18-item self-assessment for healthcare providers. All LGBT-DOCSS items are 7-point Likert scales (1 = strongly disagree to 7 = strongly agree), eight of which are reverse scored. The “Overall LGBT-DOCSS” is the overall mean score of all items, while “Clinical Preparedness”, “Attitudinal Awareness”, and “Basic Knowledge” are subscale average scores pertaining to select items (Table 2). Higher scores denote higher levels of clinical preparedness and knowledge and more positive attitudes regarding LGBT healthcare. As the original analyses of the LGBT-DOCSS did not specify cutoffs for competency level, the highest 1-point stratification (i.e., scores of 6 or more) was a priori considered “high” competency, scores of 5 to 6 were considered “moderate” competency, and scores lower than 5 were considered “low” competency. While the LGBT-DOCSS has not been applied to medical students broadly, its interdisciplinary utility for medical students is promising as the initial analyses of the LGBT-DOCSS included some medical students.

**Table 2:** LGBT-DOCSS^a^ score means

Clinical Preparedness	M (SD)	Attitudinal Awareness	M (SD)	Basic Knowledge	M (SD)
I would feel unprepared talking with a LGBT client/patient about issues related to their sexual orientation and/or gender identity.^b^	4.65 (1.55)	I think being transgender is a mental disorder.^b^	6.24 (1.34)	I am aware of institutional barriers that may inhibit transgender people from using health care services.	5.49 (1.29)
I have received adequate clinical training and supervision to work with transgender clients/patients.	3.07 (1.55)	A same sex relationship between two men or two women is not as strong and committed as one between a man and a woman.^b^	6.72 (0.90)	I am aware of institutional barriers that may inhibit LGB people from using health care services.	5.39 (1.30)
I have received adequate clinical training and supervision to work with LGB clients/patients.	3.66 (1.69)	LGB individuals must be discreet about their sexual orientation around children.^b^	6.31 (1.20)	I am aware of research indicating that LGB individuals experience disproportionate levels of health and mental health problems compared to heterosexual individuals.	5.97 (1.24)
I have experience working with LGB clients/patients.	4.02 (1.85)	When it comes to transgender individuals, I believe they are morally deviant.^b^	6.65 (0.98)	I am aware of research indicating that transgender individuals experience disproportionate levels of health and mental problems compared to cisgender individuals.	6.07 (1.22)
I feel competent to assess a person who is LGB in a therapeutic setting.	4.37 (1.68)	The lifestyle of a LGB individual is unnatural or immoral.^b^	6.52 (1.23)		
I feel competent to assess a person who is transgender in a therapeutic setting.	3.80 (1.64)	People who dress opposite to their biological sex have a perversion.^b^	6.64 (0.94)		
I have experience working with transgender clients/patients.	3.19 (1.91)	I would be morally uncomfortable working with a LGBT client/patient.^b^	6.72 (0.85)		
Total	3.82 (1.25)		6.54 (0.86)		5.73 (1.01)

Abbreviations: *LGBT* lesbian, gay, bisexual, and transgender; *DOCSS* Development of Clinical Skills Scale; *LGB* lesbian, gay, and bisexual

^a^Scores are averages on 7-point Likert scales (1 = strongly disagree, 4 = somewhat agree/disagree, 7 = strongly agree); for the Overall LGBT-DOCSS: M = 5.30, SD = 0.72

^b^Scores are not original; they are reverse scored per scoring instructions

### Procedure

As participation was voluntary and anonymous, this study was granted exemption by the Indiana University Institutional Review Board (IRB, Protocol #1903093806), University of Michigan IRB (Protocol #HUM00166371), and University of Washington IRB (Protocol #STUDY00007926). The survey was distributed by email to local contacts within each medical school at three different universities across the United States. Contacts were requested to forward the study survey to their respective medical student body. One reminder message was sent following initial survey distribution. Responses were collected between July and December 2019.

### Analysis

All analyses were done using SPSS Statistics 26. The number of LGBT extracurricular hours (“LGBT extracurricular hours”), i.e., hours that were not curricular, was calculated by subtracting LGBT curricular hours from LGBT total hours. Annual values of LGBT experiential variables, such as patients, curricular hours, and extracurricular hours, were computed by dividing these variables by level of training. Internal consistency Cronbach’s alpha coefficients were determined for each LGBT-DOCSS scale. Frequencies and means of demographics, experiential variables, and LGBT-DOCSS scores and individual items were calculated. Paired sample t-tests were analyzed to evaluate LGBT-DOCSS score differences as well as LGBT subgroup clinical perceptual differences. Multiple linear regression models for LGBT-DOCSS scores with demographic and experiential variables as predictors were performed. Experiential variable means were then computed per each 1-point LGBT-DOCSS score stratification, and Spearman’s rank correlation coefficients between these means and increments were determined. Medical students were divided by their cultural competency scores, i.e. those with scores of 6 or more on the Overall LGBT-DOCSS (“higher-competent” medical students) from those with scores of less than 6, experiential variable means of the higher-competent medical students were used as group splits, and analyses of covariance (ANCOVAs) were analyzed. Quotes from medical students were chosen to emphasize ideas such as patient exposure, education, and clinical perceptions (Supplemental Data).

## Results

### Variables

Many medical students filled out the study survey (Table 1). Overall, the response rate was 27.6% (university #1: 26.4%, university #2: 35.4%, and university #3: 24.5%). Most medical students were in their twenties, cisgender women, heterosexual, White/Caucasian, and not Hispanic or Latino. All levels of training, i.e., years one through four, were represented. A few of students were enrolled in dual degree programs and were in years five and above. They reported low to moderate numbers of LGBT patients and LGBT educational hours.

### Cultural competency scores

**Fig. 1:**
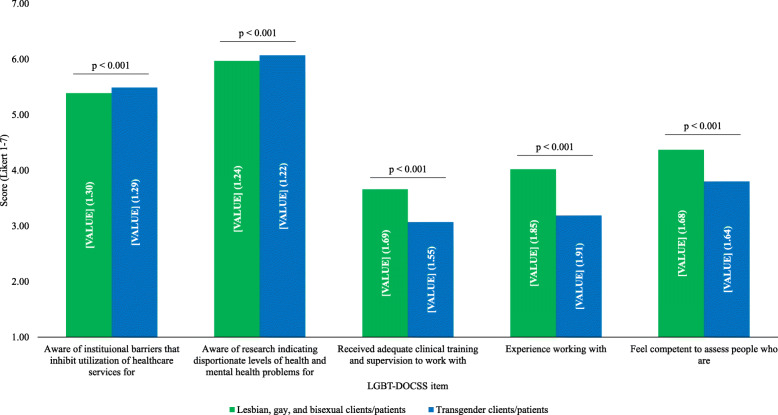
LGB vs transgender clinical perceptions. Abbreviations: *LGB* lesbian, gay, and bisexual; *LGBT* lesbian, gay, bisexual, and transgender; *DOCSS* Development of Clinical Skills Scale. LGBT-DOCSS scores are means on 7-point Likert scales. Higher scores are indicative of higher levels of clinical preparedness and knowledge and less prejudicial attitudes regarding LGBT patients. Similar LGBT-DOCSS items that differed based on patient type (i.e., LGB vs transgender) were analyzed using paired sample t-tests to determine whether there were clinical perceptual differences between LGBT subpopulations. While medical students reported significantly more awareness about institutional barriers [t (939) = 4.674] and healthcare disparities [t (939) = 3.524] that transgender patients face compared to LGB patients, they reported significantly less adequate clinical training and supervision [t (939) = − 16.652], experience [t (939) = − 18.457], and competence [t (939) = − 17.716] to assess transgender patients compared to LGB patients

Internal consistency coefficients were good (Overall LGBT-DOCSS: *a* = 0.84, Clinical Preparedness: 0.86, Attitudinal Awareness: 0.90, and Basic Knowledge: 0.81). Medical students reported a moderate Overall LGBT-DOCSS mean score (Table 2). They reported significantly higher Attitudinal Awareness compared to Basic Knowledge [t (939) = 22.505, *p* < 0.001] and Clinical Preparedness [t (939) = 56.895, *p* < 0.001]; they likewise reported significantly higher Basic Knowledge than Clinical Preparedness [t (939) = 42.766, *p* < 0.001]. Regarding LGBT subgroup perceptual differences, there was significantly more awareness about institutional barriers and healthcare disparities that transgender patients face compared to LGB patients, and there was significantly less adequate clinical training and supervision, experience, and competence to assess transgender patients compared to LGB patients (Fig. 1).

### Variable predictors of cultural competency

For all LGBT-DOCSS scores, there were significant multiple linear regression models: Overall LGBT-DOCSS [F (10, 848) = 27.298, *p* < 0.001, *R*^2^ = 0.244, significant predictors: LGBT patients, LGBT curricular hours, LGBT extracurricular hours, gender identity, sexual orientation, university, and level of training], Clinical Preparedness [F (10, 848) = 41.379, *p* < 0.001, *R*^2^ = 0.328, significant predictors: LGBT patients, LGBT curricular hours, gender identity, sexual orientation, race, and level of training], Attitudinal Awareness [F (10, 848) = 10.339, *p* < 0.001, *R*^2^ = 0.109, significant predictors: gender identity, sexual orientation, ethnicity, and university], and Basic Knowledge [F (10, 848) = 8.358, *p* < 0.001, *R*^2^ = 0.094, significant predictors: LGBT curricular hours, LGBT extracurricular hours, gender identity, sexual orientation, and university].

### Variables across cultural competency levels

**Fig. 2:**
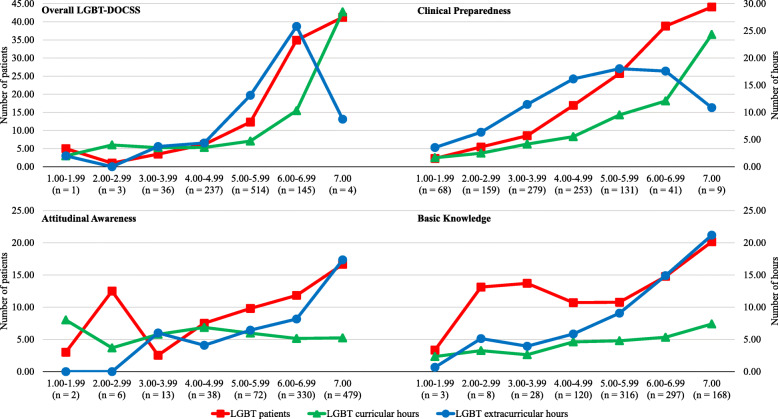
LGBT experientials across LGBT-DOCSS stratifications. Abbreviations: *LGBT* lesbian, gay, bisexual, and transgender; *DOCSS* Development of Clinical Skills Scale. LGBT-DOCSS scores are means on 7-point Likert scales. Higher scores are indicative of higher levels of clinical preparedness and knowledge and less prejudicial attitudes regarding LGBT patients. LGBT-DOCSS scores were stratified by 1-point increments and means of experiential variables (number of LGBT patients, LGBT curricular hours, and LGBT extracurricular hours) were computed per each stratification to characterize medical students with higher scores from those with lower scores. Spearman’s rank correlation coefficients were calculated to assess associations between stratifications and experiential variables: Overall LGBT-DOCSS (LGBT patients: 0.429, LGBT curricular hours: 0.281, LGBT extracurricular hours: 0.321); Clinical Preparedness (LGBT patients: 0.507, LGBT curricular hours: 0.435, LGBT extracurricular hours: 0.212); Attitudinal Awareness (LGBT patients: 0.108, LGBT curricular hours: -0.093, LGBT extracurricular hours: 0.196); and Basic Knowledge (LGBT patients: 0.131, LGBT curricular hours: 0.132, LGBT extracurricular hours: 0.257). All Spearman correlations were *p* < 0.001 except Attitudinal Awareness (LGBT curricular hours: *p* < 0.01). In general, medical students who reported higher LGBT-DOCSS scores had cared for more LGBT patients and had received more LGBT curricular and extracurricular hours of education

**Fig. 3:**
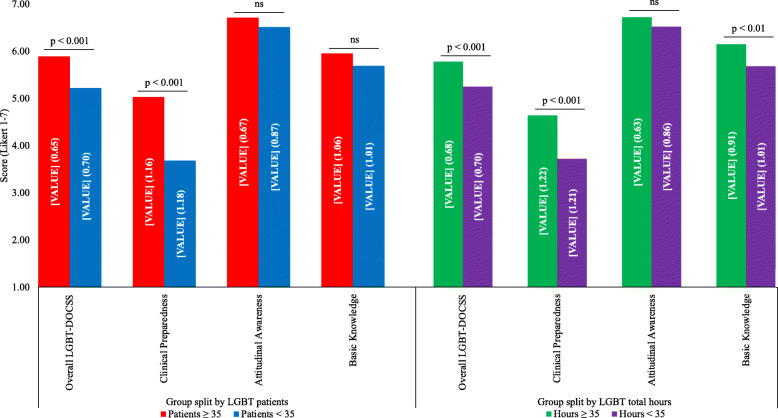
LGBT-DOCSS scores split by LGBT experientials. Abbreviations: *LGBT* lesbian, gay, bisexual, and transgender; *DOCSS* Development of Clinical Skills Scale. LGBT-DOCSS scores are means on 7-point Likert scales. Higher scores are indicative of higher levels of clinical preparedness and knowledge and less prejudicial attitudes regarding LGBT patients. For medical students with scores of 6 or more (“higher-competent” medical students) on the Overall LGBT-DOCSS, their experiential variable means of LGBT patients (i.e., 35 patients) and LGBT total hours (i.e., 35 h) served as group splits. There were significant differences of the patient split on LGBT-DOCSS scores, while adjusting for age, LGBT curricular hours, LGBT extracurricular hours, gender identity, sexual orientation, race, ethnicity, level of training, and university. There were significant differences of the hour split on LGBT-DOCSS scores, while adjusting for age, LGBT patients, gender identity, sexual orientation, race, ethnicity, level of training, and university. Medical students who had cared for 35 or more LGBT patients (*n* = 84) reported significantly higher Overall LGBT-DOCSS [F (1, 840) = 21.351] and Clinical Preparedness [F (1, 840) = 32.899] than those who cared for less than 35 LGBT patients (*n* = 787). Medical students who received 35 or more LGBT total hours (*n* = 102) reported significantly higher Overall LGBT-DOCSS [F (1, 843) = 17.154], Clinical Preparedness [F (1, 843) = 20.636], and Basic Knowledge [F (1, 843) = 7.118] than those who received less than 35 LGBT total hours (*n* = 824)

Experiential variable means tended to increase across 1-point LGBT-DOCSS score stratifications (Fig. 2). Higher LGBT-DOCSS scores were associated with more LGBT patients and LGBT educational hours. Higher-competent medical students cared for approximately 35 LGBT patients and received 35 LGBT total hours. After adjusting for demographic and experiential variables, medical students who cared for 35 or more LGBT patients and/or received 35 or more LGBT total hours had significantly higher LGBT-DOCSS scores (Fig. 3).

## Discussion

This study examines medical students’ involvement in LGBT healthcare education across multiple centers and, to our knowledge, is the first to assess how demographics, patient exposure, and curricular education influences LGBT preparedness, attitudes, and knowledge. We found that medical students reported very high attitudinal awareness, moderate knowledge, and low clinical preparedness. These findings are similar to prior studies that showed high affirming attitudes and moderate knowledge among medical students [9, 11]. Obedin-Maliver et al. [12] demonstrated that in 2009–2010 medical schools across the nation delivered a median of five total hours of LGBT-related curricular instruction during four years of medical education. From that research, it was apparent that LGBT-related medical education was not only scant but also highly variable among U.S. medical institutions. Our finding of 2.22 annual hours of LGBT-related education is only a modest improvement from the five total curricular hours reported by Obedin-Maliver et al. nearly 10 years ago.

Importantly, we found that as medical students cared for more LGBT patients and received more LGBT education, they reported higher LGBT-DOCSS scores. This finding is akin to the few studies that have shown that for medical students, LGBT patient contact and curricular education can be effective in increasing comfort [6], knowledge [6], and confidence [7] in caring for the LGBT population. However, to our knowledge, there are no specific recommendations regarding the amount of patient exposure and education that relate to high LGBT cultural competency. As educators and curricular leadership often experience competing demands for increased educational hours on a number of topics, we aimed to quantify the specific patient encounter and curricular hour benchmarks that could be recommended to promote high LGBT cultural competency. By setting a high, yet reasonable, standard for proficiency, we found that medical students with high cultural competency (i.e., those who reported an Overall LGBT-DOCSS score near 6) cared for 35 or more LGBT patients and received 35 or more LGBT total education hours. The effect of LGBT patient exposure and education on cultural competency was most apparent in Clinical Preparedness, which had the largest difference in scores.

Of the 35 curricular hours that higher-competent medical students received, 25 of those hours were extracurricular, suggesting that many medical students may heavily rely on self-directed LGBT education. While this finding could indicate that incorporating 10 LGBT curricular hours may be stimulating enough for medical students to pursue supplementary education to achieve a total of 35 LGBT hours, it is also problematic. Nearly 30% of medical students in this study did not report any extracurricular education and thus relied exclusively on their programs for their LGBT education. As such, a large proportion of medical students may not achieve 35 h if these hours are not provided explicitly by their programs.

To close the current gap of nationally inadequate LGBT cultural competency, medical schools should consider an LGBT educational curriculum that consists of approximately nine annual hours (both 2.5 h of required curricular education and 6.5 h of supplemental education) over the course of the typical four-year medical education timeline. For schools without any or minimal integration of LGBT topics and patients, an LGBT educational curriculum could easily be delivered and required within lectures, case presentations, and small group sessions across different courses and levels of training; additional, supplemental, non-required education could be encouraged through online modules, journal clubs, seminars, conferences, and clinical rotations such as multidisciplinary and sexual and gender minority clinics and electives. An interesting inquiry is how much impact this amount of curricular and supplemental education has on subsequent clinical performance, patient-provider shared decision making, patient satisfaction, and patient outcomes.

With regards to the transgender population, the lowest item mean of the entire LGBT-DOCSS addressed having received adequate clinical training and supervision to work with transgender patients. Additionally, we found a disconnect in medical students’ moderately high reported understanding of transgender-specific knowledge and their very low reported transgender-specific preparedness, especially when compared to their preparedness in treating LGB patients. This discomfort with transgender care specifically has been described in prior studies among medical students [10, 11] and may imply that there is a lack of educational emphasis on transgender-related topics, particularly those that involve clinical preparedness. Dubin et al. [15] noted curricular time as a barrier to transgender health exposure. Taken together with our findings, not only should LGBT education as a whole be increased, but special attention should be given to transgender-related healthcare topics. As one surveyed student conveyed, “While frequently spoken of as one group, I feel that treating LGB patients and transgender patients are two entirely different experiences.”

Future studies are required to: 1) examine the specific proportion of transgender-specific patient exposure and hours which relate to high transgender cultural competency, 2) examine the long-term effects that increased LGBT patient exposure and curricular education have on LGBT cultural competency, clinical performance, and patient outcomes, and 3) recommend standardized, universal cultural competency training for medical students. Also, given the spectrum of gender identities, sexual orientations, races, and levels of training, this sample population represents a diverse pool of medical students, although with notably more cisgender women and White/Caucasian medical students than the entire medical student population [16] as well as notably more sexual and gender minorities than the general U.S. population [17]. While not the specific aim of this study, these demographic variables are important attributes to consider in future research, as many were significant predictors of LGBT-DOCSS scores. Future LGBT cultural competency studies should consider incorporating more sexual, gender, and racial minority medical students by direct outreach to diversity student groups as well as diversity and inclusion offices at medical institutions.

Study limitations do exist. Firstly, this study was conducted at only three universities. In addition, reliance on convenience sampling may have caused medical students with biases toward the LGBT population to not participate in this study. Taken together, the degree of generalizability of these results to the national medical student population is unknown. Secondly, the accuracy of the self-reported experiential variables is unknown. Thirdly, self-reported quality of LGBT educational hours was not assessed. Lastly, only 50 medical students were polled in the initial validity analyses of the LGBT-DOCSS [14], and a broader use of the LGBT-DOCSS within medical student populations has only been presented here.

## Conclusions

This study examines 940 medical students’ LGBT cultural competency at three institutions across the country. Our data highlights the positive attitudes of medical students but also identifies their self-reported inadequacies in LGBT patient care-related clinical skills and preparedness. Currently, at the institutional level, there is limited LGBT patient exposure and education given to medical students. In striving to improve medical students’ LGBT cultural competency, medical schools and accrediting bodies such as the Liaison Committee of Medical Education should consider providing medical students with least a total of 35 LGBT patient contacts and 35 LGBT education hours (10 h of required curricular education and 25 h of supplemental education). Additionally, special emphasis should be given to transgender-related patient care topics.

## Supplementary Information

**Additional file 1.**

## Data Availability

The datasets used and/or analyzed during the current study are available from the corresponding author on reasonable request.

## Abbreviations

LGBT: Lesbian, gay, bisexual, and transgender
DOCSS: Development of Clinical Skills Scale
