# Correction: How much is needed? Patient exposure and curricular education on medical students’ LGBT cultural competency

**DOI:** 10.1186/s12909-022-03483-8

**Published:** 2022-06-07

**Authors:** Dustin Z. Nowaskie, Anuj U. Patel

**Affiliations:** 1 Department of Psychiatry, Indiana University School of Medicine, 355 W. 16th St, #2364, Indianapolis, IN 46202 USA; 2 University of Michigan Medical School, Ann Arbor, MI USA

**Correction: BMC Med Educ 20, 490 (2020)**

**https://doi.org/10.1186/s12909-020-02381-1**

Following publication of the original article [1], the authors identified an error in Figs. 1 and 3. The correct figures are given below.

**Fig. 1:**
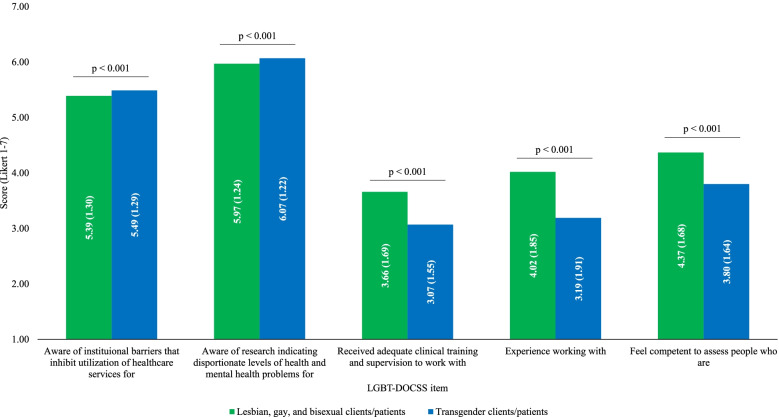
LGB vs transgender clinical perceptions. Abbreviations: LGB lesbian, gay, and bisexual; LGBT lesbian, gay, bisexual, and transgender; DOCSS Development of Clinical Skills Scale. LGBT-DOCSS scores are means on 7-point Likert scales. Higher scores are indicative of higher levels of clinical preparedness and knowledge and less prejudicial attitudes regarding LGBT patients. Similar LGBT-DOCSS items that differed based on patient type (i.e., LGB vs transgender) were analyzed using paired sample t-tests to determine whether there were clinical perceptual differences between LGBT subpopulations. While medical students reported significantly more awareness about institutional barriers [t (939) = 4.674] and healthcare disparities [t (939) = 3.524] that transgender patients face compared to LGB patients, they reported significantly less adequate clinical training and supervision [t (939) = − 16.652], experience [t (939) = − 18.457], and competence [t (939) = − 17.716] to assess transgender patients compared to LGB patients

**Fig. 3:**
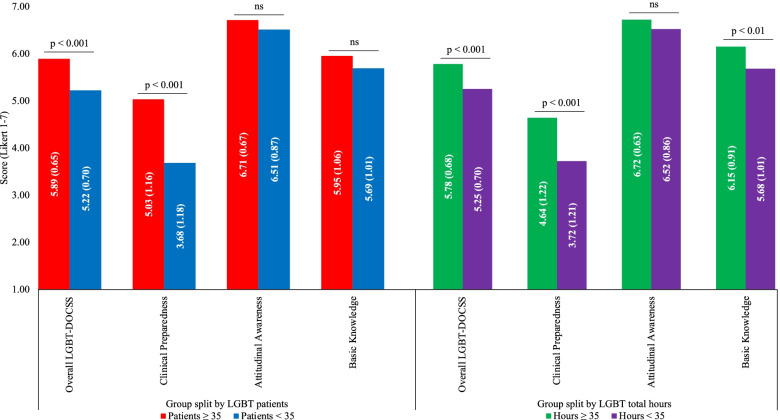
LGBT-DOCSS scores split by LGBT experientials. Abbreviations: LGBT lesbian, gay, bisexual, and transgender; DOCSS Development of Clinical Skills Scale. LGBT-DOCSS scores are means on 7-point Likert scales. Higher scores are indicative of higher levels of clinical preparedness and knowledge and less prejudicial attitudes regarding LGBT patients. For medical students with scores of 6 or more (“higher-competent” medical students) on the Overall LGBT-DOCSS, their experiential variable means of LGBT patients (i.e., 35 patients) and LGBT total hours (i.e., 35 h) served as group splits. There were significant differences of the patient split on LGBT-DOCSS scores, while adjusting for age, LGBT curricular hours, LGBT extracurricular hours, gender identity, sexual orientation, race, ethnicity, level of training, and university. There were significant differences of the hour split on LGBT-DOCSS scores, while adjusting for age, LGBT patients, gender identity, sexual orientation, race, ethnicity, level of training, and university. Medical students who had cared for 35 or more LGBT patients (n = 84) reported significantly higher Overall LGBT-DOCSS [F (1, 840) = 21.351] and Clinical Preparedness [F (1, 840) = 32.899] than those who cared for less than 35 LGBT patients (n = 787). Medical students who received 35 or more LGBT total hours (n = 102) reported significantly higher Overall LGBT-DOCSS [F (1, 843) = 17.154], Clinical Preparedness [F (1, 843) = 20.636], and Basic Knowledge [F (1, 843) = 7.118] than those who received less than 35 LGBT total hours (n = 824)

The original article has been corrected.
