# Supplementary material for: How much is needed? Patient exposure and curricular education on medical students’ LGBT cultural competency
Source: BMC Med Educ. 2020 Dec 4;20:490. doi: 10.1186/s12909-020-02381-1 (PMC7716501; doi:10.1186/s12909-020-02381-1)
Supplement: Supplementary file 1 — Additional file 1. [file 12909_2020_2381_MOESM1_ESM.docx]

| Supplemental Data. Medical students’ quotes across LGBT cultural competency theme | | |
| --- | --- | --- |
| LGBT patient exposure | LGBT education | LGB vs transgender clinical perceptions |
| *I think exposure is the biggest thing that could help me. It is easy to just put up PowerPoint slides and not think about it, but actually meeting a person and learning about them could improve care.*  *I think it should be covered more in healthcare education curricula. I think this is best done through first-hand experience with patients who identify as LGBTQ, rather than from learning from a module or watching videos.*  *I think some of this learning will come with experience, which can't really be taught/manufactured through 'trainings'. I'm sure I will have awkward experiences and say the wrong thing or not ask the right questions. Hopefully I will learn to be okay with this instead of turning away from the anxiety/awkwardness this causes me.*  *I wish I knew more about it and I really wish I had more exposure to working with this community. I feel grossly unprepared to work for and with LQBTQ community.*  *I think when our own experiences are different, it’s hard to know all the issues a patient is dealing with without speaking directly to the affected patients and listening. So, while our program can teach us how to establish a safe space, listen, ask questions–a large part of our experience and learning comes from the patients themselves.* | *I would like more education! I am very supportive and accepting of all patients, but LGBT care is something that can be intimidating in the moment. I understand that these individuals have various barriers to care and really want to care for them in a way that is truly personal and patient-centered, but I think that takes greater understanding and education regarding the LGBT community and the ways their healthcare needs may be different from cisgender individuals.*  *LGBTQ healthcare should NOT be an elective; instead, it should be integrated throughout the medical school curriculum and be mandatory for all students.*  *Much of what I know about barriers that LGBTQ people face in healthcare I've learned from conversations with friends who identify as members of this community and my own research due to desire to learn more. [My school] has not at all prepared me to provide appropriate care to this population. I've had to teach myself.*  *Not only is education currently inadequate at almost all med schools, workshops allowing current practitioners to recognize and work past their biases is severely inadequate and seems to be deemed as less important or a lost cause—a lot of continued training in this area is viewed as optional and not essential.*  *Students often have to go out of their way to gain additional training for LGBTQ healthcare rather than it being part of the standard curriculum for all students.* | *I feel very comfortable with LGB patients but have much more limited exposure to trans patients. Things like when to ask what pronouns to use (or how to handle accidentally using the wrong pronouns) when first meeting someone are not intuitive to me and I could use some education on that.*  *When filling out this survey, for some reason I found myself categorizing lesbian/gay/bisexual individuals differently than transgender individuals. Perhaps it’s because I have more experience working with LGB patients than transgender patients, but maybe it is also an implicit bias.*  *While frequently spoken of as one group, I feel that treating LGB patients and transgender patients are two entirely different experiences.* |
